# Peripheral blood gene expression: it all boils down to the RNA collection tubes

**DOI:** 10.1186/1756-0500-5-1

**Published:** 2012-01-04

**Authors:** Andreas Menke, Monika Rex-Haffner, Torsten Klengel, Elisabeth B Binder, Divya Mehta

**Affiliations:** 1 Max Planck Institute of Psychiatry, Kraepelinstr. 10, D-80804 Munich, Germany

## Abstract

**Background:**

Gene expression profiling from peripheral blood is a valuable tool for biomarker discovery in clinical studies. Different whole blood RNA collection and processing methods are highly variable and might confound comparisons of results across studies. The main aim of the study was to compare genome-wide gene expression profiles obtained from the two widely used commercially available whole blood RNA collection systems - PAXgene™ and Tempus™ tubes. Comparisons of present call rates, variances, correlations and influence of globin reduction across the two collection systems was performed using *in vivo *glucocorticoid stimulation in 24 peripheral blood samples from three individuals.

**Results:**

RNA quality, yield and numbers of detected transcripts from the two RNA collection systems was comparable, with no significant differences between the tube types. Globin reduction resulted in a significant increase in present call rates (*p *= 8.17 × 10^-5 ^and *p *= 1.95 × 10^-3 ^in PAXgene™ and Tempus™ tubes respectively) and significant decrease in gene expression variance in both RNA collection tubes (*p *= 0.0025 and *p *= 0.041 in PAXgene™ and Tempus™ tubes respectively). Comparisons of glucocorticoid receptor-stimulated gene expression profiles between the two collection tube systems revealed an overlap of only 17 to 54%, depending on the stringency level of the statistical thresholds. This overlap increased by 1-8% when the RNA samples were processed to remove the globin mRNA.

**Conclusion:**

RNA obtained from PAXgene™ and Tempus™ tubes was comparable in terms of quality and yield, however, detectable gene expression changes after glucocorticoid receptor stimulation were distinct, with an overlap of only up to 46% between the two collection systems. This overlap increased to 54% when the samples were depleted of globin mRNA and drastically reduced to 17-18% when only gene expression differences with a fold change greater than 2.0 were assessed. These results indicate that gene expression profiles obtained from PAXgene™ and Tempus™ differ drastically and should not be analyzed together. These data suggest that researchers must exert caution while interpreting expression profiles obtained through different RNA collection tubes.

## Background

Microarray gene expression profiling is a frequently used approach to identify susceptibility genes or biomarkers for a number of human diseases. Peripheral blood represents a target tissue not only for blood-borne diseases, but it is also an attractive surrogate tissue for other diseases, given that peripheral blood cells share more than 80% of the transcriptome with many tissues ranging from brain, colon, heart, kidney, lung, prostate to spleen and stomach [1]. Whole blood is a valuable resource which is easily obtainable and from which RNA can be prepared economically with standardized extraction protocols. Genome-wide expression profiling of human blood samples however, faces numerous challenges. Robust detection of transcript levels and high reproducibility in conjunction with minimal experimental variance is essential to enable detection of true biological variance. While standardization of blood sample handling and processing procedures is essential for better comparisons of gene expression results across experiments, very few studies have investigated the influence of sample collection and its impact on whole blood transcriptome analysis.

Two commercial systems are available for collection and immediate stabilization of peripheral blood RNA: the PAXgene™ Blood RNA Systems (PreAnalytiX) and Tempus™ Blood RNA Tubes (Applied Biosystems). These RNA collection systems use proprietary reagents which lyse blood cells immediately after collection in the tubes, stabilize the RNA and prevent induction of new transcripts. In addition to different RNA collection methods, there is also a current debate whether high amounts of globin mRNA, originating from immature reticulocytes reduces the sensitivity to detect differently expressed genes and subsequently adversely impact gene expression profiling using microarrays [2]. While globin reduction appeared to be beneficial in terms of decreased variability in gene expression profiles and detection of small differences using Affymetrix arrays [3], no increased sensitivity in the detection of transcriptional changes was observed using the Applied Biosystems Microarray System [4]. A technical note also suggests no benefits of globin reduction when using Illumina BeadChip expression microarrays [5]. Finally, RNA processing approaches in combination with globin reduction may produce distinct gene expression signatures which might not be directly comparable to expression profile from RNA without globin reduction [4,6].

To address the concerns about the reproducibility and robustness of peripheral blood RNA collection systems, we compared gene expression profiles from RNA samples collected in PAXgene™ and Tempus™ tubes using Illumina BeadChips microarrays. Since peripheral blood gene expression is known to be influenced by various factors including age, gender, subset of leucocytes, body mass index, and smoking, all of which increase the biological variability, we performed an *in vivo *challenge test to override or at least reduce these confounding factors. In previous studies glucocorticoids have been shown to induce up- or down-regulation of 100-1,000 genes in a cell-type specific way [7] hence we used dexamethasone, a synthetic cortisol analogon, selective for the glucocorticoid receptor, to stimulate gene expression levels. The *in vivo *challenge test enabled comparisons between glucocorticoid receptor stimulated changes across the two different RNA processing methods.

## Results

### RNA yield and quality comparisons between PAXgene™ and Tempus™ tubes

Differences between the two RNA collection systems were assessed at the level of total RNA yield, RNA quality measured by the spectrophotometer ratio and the RNA Integrity Number (RIN) was assessed using an Agilent 2,100 Bioanalyzer [8]. High quality RNA was obtained from both PAXgene™ and Tempus™ tubes with average 260/280 ratios of 2.1 and an average RIN of 8.7 for PAXgene™ and 8.2 for Tempus™ tubes. Following globin reduction, there was a significant reduction in the RIN in both tubes (PAXgene™ average RIN - 8.2, p-value: 4.9 × 10^-03 ^and Tempus™ average RIN - 7.1, p-value: 4.0 × 10^-05^). The total RNA yield obtained from the PAXgene™ samples was significantly lower than that obtained with Tempus™ tubes (2.04 μg/ml blood ± 0.76 versus 2.98 ± 0.91 μg/ml, *p *= 0.022). Globin reduction led to a loss of 47.3 ± 14.7% in PAXgene™ tubes and of 27.0% ± 7.8% of the RNA in Tempus™ tubes.

### Comparisons of baseline RNA expression between PAXgene™ and tempus™ tubes

#### Without globin reduction

The number of significantly detected transcripts was determined using the Illumina probe detection threshold of *p *≤ 0.01 in all samples within the group. There were no significant differences in the present call rates between the two RNA collection systems (*p *= 0.158). Overall, the mean number of transcripts detected above this threshold in PAXgene™ tubes was 8870 ± 197 and in Tempus™ tubes was 9265 ± 143. The average Pearson correlation between expression levels from the two systems was 0.94. No significant difference in the mean variance between expression profiles obtained from the PAXgene™ (3.03) versus Tempus™ (3.12) tubes was observed (*p *= 0.090). Baseline differences for two transcripts, TOMM7 and RAB37, whose expression levels were significantly different between the PAXgene™ and Tempus™ tubes were validated using qPCR (Additional file 1: Figure S1).

#### After globin reduction

After globin-reduction, a significant increase in the number of probes with expression signals above detection threshold was observed in both PAXgene™ and Tempus™ tubes (Average number of significant detected genes in PAXgene™ tubes and Tempus™: pre-globin reduction - 9762 ± 972 and 9918 ± 482, post-globin reduction - 11480 ± 401 and 11608 ± 247, *p *= 8.17 × 10^-5 ^and *p *= 1.95 × 10^-3 ^in PAXgene™ and Tempus™ tubes, respectively). An average correlation of R = 0.97 was observed for the 7,578 transcripts significantly detected in the pre- versus post-globin reduced samples. In accordance with previous reports [6,9], globin reduction resulted in a significant reduction in the variance (PAXgene™ tubes: mean variance of pre-globin reduced samples - 3.20, mean variance of post-globin reduced samples - 2.89, *p *= 0.0025 and Tempus™ tubes: mean variance of pre-globin reduced samples - 3.21, mean variance of post-globin reduced samples - 2.94, *p *= 0.041).

### Comparison of PAXgene™ RNA and tempus™ collection systems in combination with globin reduction on dexamethasone stimulated gene expression profiles

**Figure 1:**
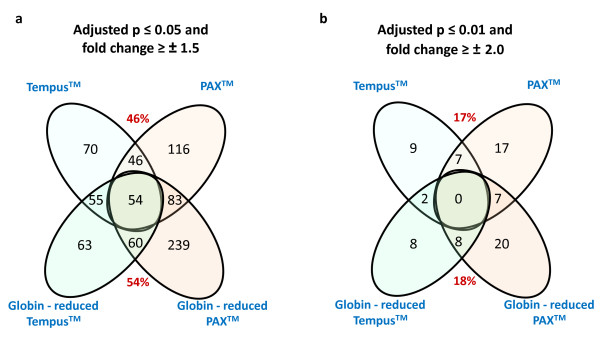
**Venn diagram comparing PAXgene™ and Tempus™ tube gene expression: Comparisons based on dexamethasone stimulated gene expression before and after globin-reducing the RNA**. Differentially regulated genes withstanding correction for multiple testing are shown. In red are the percentages of overlap between the PAXgene™ and Tempus™ tubes. a) Comparisons using the less stringent statistical filter of adjusted *p *< 0.05 and fold change of ≥ ± 1.5. b) Comparisons using the more stringent statistical filter of adjusted *p *< 0.01 and fold change of ≥ ± 2.0.

After dexamethasone challenge 11107 ± 930 transcripts were detected in PAXgene™ samples and 10135 ± 1500 in Tempus™ specimens before globin reduction, with no significant increase in the present call rates (*p *= 0.25). To test the reproducibility between expression profiles obtained from the PAXgene™ and Tempus™ tubes, linear regression models were used to analyze transcripts that were differentially regulated after dexamethasone stimulation in the pre-globin depleted samples. To avoid a bias due to different detection rates between the two systems both before and after globin reduction and to ensure analysis of only robustly expressed transcripts, we analyzed 8,263 probes which were significantly detected in all 24 samples. Two arbitrary thresholds of statistical significance were set to determine which transcripts were significantly regulated after dexamethasone intake: adjusted *p *≤ 0.05 and fold change of ≥ ± 1.5 and adjusted *p *≤ 0.01 and fold change of ≥ ± 2.0. At adjusted *p *≤ 0.05, 449 transcripts and 365 transcripts were significantly regulated with fold changes of ≥ ± 1.5 after dexamethasone stimulation in the PAXgene™ and Tempus™ tubes, respectively (Figure 1). An overlap of 100 transcripts between the two systems corresponding to a 46% overlap was observed. At adjusted *p *≤ 0.01, 31 transcripts and 18 transcripts were significantly regulated with fold changes of ≥ ± 2.0 after dexamethasone stimulation in the PAXgene™ and Tempus™ tubes respectively (Figure 1). An overlap of 7 transcripts between the two systems corresponding to a 17% overlap was observed.

Since comparisons between the PAXgene™ and Tempus™ tubes might be confounded by varying amounts of globin mRNA, the analysis was repeated using the globin depleted samples. Overall, there was no significant difference in the number of significant dexamethasone regulated genes after globin reduction in the PAXgene™ and Tempus™ tubes (*p *= 0.996). In the globin-depleted samples, 550 transcripts and 232 transcripts were significantly regulated after dexamethasone stimulation at adjusted *p *≤ 0.05 and 35 transcripts and 18 transcripts were significantly regulated after dexamethasone stimulation in the PAXgene™ and Tempus™ tubes, respectively at adjusted *p *≤ 0.01 (Figure 1). An overlap of 114 transcripts between the two systems corresponding to a 56% overlap was observed at adjusted *p *≤ 0.05 and an overlap of 8 transcripts corresponding to a 18% overlap was seen at adjusted *p *≤ 0.01 (Figure 1).

For FKBP5 and IL18R1, we validated the results using qPCR to demonstrate the technical reliability of these data. The average correlation of the delta Ct against the normalized microarray expression levels for FKBP5 was 0.914 (*p *= 4.15e-07) and that for IL18R1 was 0.834 (*p *= 4.38e-03). Using qPCR, we observed a significant up regulation (*p *< 0.05) of both FKBP5 and IL18R1 following dexamethasone stimulation in all conditions (PAXgene™ and Tempus™ tubes both with and without globin reduction) and as suggested by the high correlations coefficients, the qPCR fold changes for all conditions were highly comparable with those obtained by microarray. For instance for Tempus™ tubes the mean fold change [standard error of mean] and p-value for FKBP5 were as follows - microarrays before globin reduction: 5.53, *p *= 0.022 and qPCR before globin reduction: 5.82 [± 1.13], *p *= 0.045; microarrays after globin reduction: 5.92, *p *= 0.032 and qPCR after globin reduction: 6.12 [± 0.33], *p *= 0.030.

Preliminary pathway enrichment analysis was performed using Wikipathways (http://bioinfo.vanderbilt.edu/webgestalt/) to identify pathways which were significantly over-represented in the PAXgene™ versus Tempus™ tubes after Bonferroni corrections for multiple testing. Among the 54 transcripts which were significantly induced by dexamethasone stimulation under all conditions (at adjusted *p *≤ 0.05 and fold change of ≥ ± 1.5), there was a significant over-representation of the Toll receptor, adipogenesis, T-cell receptor, IL-7 and IL-2 pathways, which have previously reported to be glucocorticoid sensitive pathways [10]. In addition, there was a selective enrichment of DNA damage response and cell cycle pathways in Tempus™ tubes only, while Translation factors, pentose phosphate, TGF-beta receptor and Hedgehog signal-ling pathways were enriched in PAXgene™ tubes only.

## Discussion

To assess whether PAXgene™ and Tempus™ peripheral blood RNA collection tubes provide comparable gene expression profiles, we performed an *in vivo *challenge test with glucocorticoids in healthy volunteers to assess dynamic changes in gene expression profiles.

The PAXgene™ and Tempus™ tubes were highly comparable in terms of RNA quality although consistent with previous studies, the RNA yield from PAXgene™ tubes was significantly lower [11]. No significant differences in the detection rates between the two RNA collection systems were observed. Expression profiles from the two tube types showed a Pearson correlation coefficient of 0.94, a value much lower than the usual Pearson correlation of 0.98-99 which is often observed for replicates [12]. The mean variance in transcriptional levels obtained from Tempus™ tubes was higher than the variance in PAXgene™ tubes but this difference was not statistically significant. The finding that globin reduction significantly increased the sensitivity and significantly reduced the overall variance in gene expression profiles from both tubes was in line with previous studies reporting the positive impact of globin reduction on whole blood gene expression [6,9].

**Figure 2:**
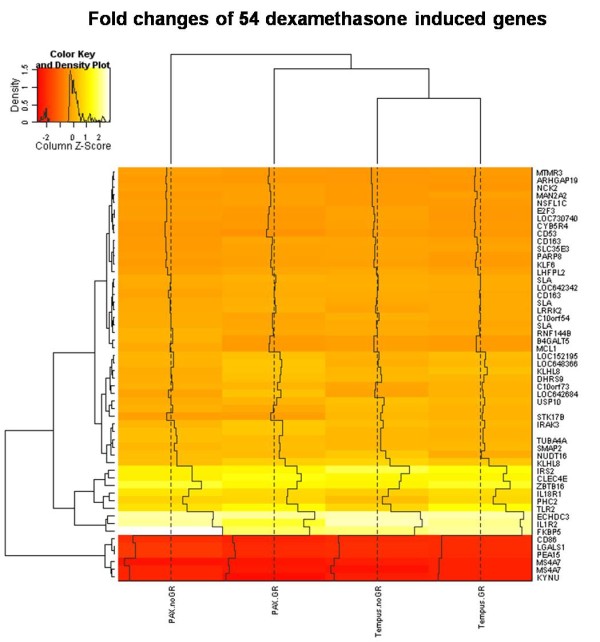
**Comparison of fold changes between PAXgeneTM and TempusTM tubes after dexamethasone-stimulation: Heatmap of fold changes of 54 significantly differentially expressed transcripts after dexamethasone-stimulation in PAXgene™ and Tempus™ tubes**. The legend displays the colour mapping to row-wise Z-scores, which are calculated by subtracting the mean from each cell, and then dividing the value by the standard deviation of the row. The density plot in the legend indicates that most transcripts have consistent fold changes across the 4 groups (non-significant differences are indicated in orange). Few transcripts show significant differences in dexamethasone induction as indicated in red and white. The histogram in the heatmap indicates the z-score scaling across the rows, with the dotted black line indicating a z-score of 0. The transcripts are ordered according to their z-score scaling across the rows, from the non-significant z-scores around 0 on the top and the significant z-scores around 2 at the bottom.

Comparisons of the PAXgene™ and Tempus™ tubes were performed using *in vivo *dexamethasone stimulation as a tool to assess the comparability of dynamic within- subject changes in gene expression between the two collection systems. Since setting the threshold of significance for statistical tests is an arbitrary measure, we set two thresholds which differed in the level of stringency. The threshold level of adjusted p < 0.05 and fold change of ≥ ± 1.5 was a less stringent filter but probably closer to the threshold used in most microarray studies where usually only nominal fold changes are expected. The more stringent threshold of adjusted p < 0.01 and fold change of ≥ ± 2.0 were chosen to select for highly robust changes in gene expression levels and to allow comparison of our results with that of Asare and colleagues [11] who have also assessed the influence of RNA collection systems on gene expression profiles. Interestingly, despite using two statistical thresholds, the expression profiles obtained from PAXgene™ and Tempus™ tubes did not substantially overlap, with 46% overlap using the less stringent statistical threshold and the overlap being reduced to only 17% using the more stringent statistical threshold. Furthermore, we observed that the differences between the PAXgene™ and Tempus™ tubes could not be overridden by globin-reduction of the samples. In fact, removal of globin-mRNA did not greatly increase the sensitivity to detect dexamethasone regulated transcripts. The overlap of 17% using the more stringent criteria showed a gain in sensitivity of only 1% over the non globin reduced samples. Of the 54 transcripts which were significantly differentially regulated after dexamethasone stimulation under all conditions using the less stringent statistical filters (Figure 2), there are several candidates such as FKBP5, IL18R1, IL1R1, ECHDC3, ZBTB16, PHC2 and TLR2 which we and others have found to be robustly induced by dexamethasone across independent datasets, thereby serving as positive controls [13,14].

To date only two studies have compared expression levels from RNA collected in PAXgene™ and Tempus™ tubes. Matheson et al. reported that there were no significant differences between IL-2 levels between the two tubes which were shipped at two different temperatures [15]. In another study, Asare and colleagues demonstrated that peripheral blood RNA isolation using the PAXgene™ and Tempus™ tubes is significantly different and this critically impacts gene expression profiles obtained from the two RNA collection systems [11]. In this study the average overlap between the two tubes for phytohemagglutinin (PHA) induced gene expression differences was 48% (at *p *< 0.01 and fold change > 2) when analyzed on the Affymetrix microarray system. In the current study we report a sizeable difference between the two tubes types with an overlap of only 17-18% on the Illumina microarray at the same statistical threshold. The discrepancy in the percentage of overlap might be partly explained by differences in the experimental set-up, type of stimulus to induce gene expression, microarray platforms and statistical procedures between the two studies.

The major disadvantage of this study is the small sample size and it would be interesting to replicate these findings independently in a larger dataset. The advantages of the study include a robust *in vivo *stimulation to detect dynamic differences in gene expression and qPCR validation of four transcripts in triplicates to demonstrate the technical reliability of the data. A further strength of this study is that the reported gene expression differences between the PAXgene™ and Tempus™ tubes seem robust to RNA purification methods such as globin reduction. Also, to the best of our knowledge, this is the only study which compares the PAXgene™ and Tempus™ tubes in the presence of globin reduction.

## Conclusions

Multicenter research studies involving collection and storage of large numbers of peripheral blood samples require a systematic approach for sample collection which is reliable and reproducible across different laboratories. Our results indicate that the choice of RNA collection tubes greatly influences the transcriptional profiles obtained from peripheral blood. While comparing transcripts differentially regulated after *in vivo *dexamethasone intake, we demonstrate a mere overlap of up to 54% between two of the most commonly used commercial systems for collection of peripheral blood RNA - PAXgene™ and Tempus™ tubes. We report that only transcripts which are differentially regulated with large, robust and significant fold changes are detected in both PAXgene™ and Tempus™ tubes. These results raise a very important question about the strong impact of the RNA isolation tubes on transcription levels which cannot be surpassed by downstream processes such as globin mRNA reduction and data analysis. These data suggest that combining gene expression profiles across the PAXgene™ and Tempus™ tubes is not advisable and researchers should use a single tube type throughout the project to avoid biases due to the RNA collection system. This study has great relevance for large clinical studies where whole blood gene expression profiles are analyzed for general research purposes and for identification of biomarkers for human diseases.

## Methods

### Study design and blood collection

**Figure 3:**
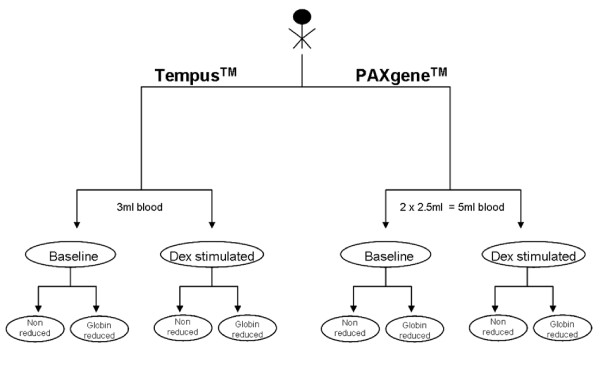
**Flow diagram of the study design: Peripheral blood was drawn from 3 healthy young male volunteers at 12:00 and 3:00 pm using PAXgene™ and Tempus™ collection tubes**. After the first blood draw at baseline, volunteers were administered 1.5 mg dexamethasone orally and blood was collected again after 3 h. After blood collection, RNA (24 samples, 8 from each individual) was extracted using standard protocols and later processed either with or without globin reduction. Blood from 2 PAXgene™ tubes was pooled together to obtain enough RNA for the amplifications and the globin reduction procedure.

Three healthy male volunteers aged 30-35 years with body mass index of 20-25 were enrolled in the study (see Figure 3). All subjects were in good physical condition and free from medication. Leucocytes counts, C-reactive protein (CRP), gamma glutamyl transpeptidase (GGT), alanine aminotransferase (ALAT) and aspartate aminotransferase (ASAT) levels were not significantly elevated. The optimal time of blood collection for mRNA measurement after dexamethasone stimulation was defined in a pilot experiment. The t_max _of orally administered dexamethasone is about 1.5 h with a t_1/2 _of about 4 h [7]. In our pilot experiment, maximal gene expression was observed 3 and 5 h after dexamethasone intake, hence we used a 3 h interval between blood sampling. Non-stimulated peripheral blood samples were obtained at noon after 2 h of fasting and abstention from coffee, smoking and physical activity, followed by orally administration of 1.5 mg dexamethasone. Stimulated peripheral blood samples were drawn 3 h later. For each sample, duplicate measures of 2.5 ml and 3 ml blood was collected in PAXgene™ tubes (PreAnalytiX GmbH, Hombrechtikon, Switzerland) and Tempus™ tubes (Applied Biosystems, Foster City, CA, USA) respectively. The PAXgene™ and Tempus™ tubes were stored for 2.5 h at room temperature and then transferred to -20°C. Due to the lower RNA yield from the PAXgene™ tubes, RNA from two PAXgene™ tubes was pooled together for the experiment.

Serum, EDTA and Li Herapin tubes (Sarstedt AG, Nümbrecht, Germany) were collected concurrently to analyse differential blood counts, cortisol and ACTH levels and liver enzymes. Administration of 1.5 mg dexamethasone p.o. resulted in a decrease in cortisol and ACTH levels at 3 h post-intake, indicating successful dexamethasone stimulation in all 3 volunteers.

The study was approved by the Ethics Committee of the Ludwig Maximilians University in Munich, Germany, and written informed consent was obtained from all subjects.

### RNA extraction, globin reduction and amplification

Total RNA was isolated from whole blood stored in PAXgene™ and Tempus™ tubes according to the respective manufacturer's instructions. The PAXgene™ samples were processed using the PAXgene™ Blood RNA Kit based on the Quiagen method for column purification of nucleic acids (PreAnalytiX GmbH, Hombrechtikon, Switzerland, catalogue number 762174). The Tempus™ samples were isolated using the Tempus™ Spin RNA Isolation Kit (Applied Biosystems, Foster City, CA, USA, catalogue number 4380204). RNA quality was assessed using an Agilent 2100 Bioanalyzer (Agilent Technologies, Palo Alto, CA, USA). The concentration and purity of total RNA was independently assessed by 260/280 UV absorption ratios, respectively (Nanophotometer, Implen, Munich, Germany). RNA samples were divided into non-globin reduced and globin reduced groups. The GLOBINclear™-Human Kit (Ambion, Inc., Texas, USA, catalogue number #AM1980) was used to remove globin mRNA. Sample amplification and labeling for both globin-reduced and non-reduced samples was performed using the Illumina^R ^TotalPrep RNA Amplification Kit (Ambion, Inc., Texas, USA, catalogue number AMIL1791).

### Processing of samples on microarrays

Biotin-labeled cRNA was hybridized to Illumina HumanHT-12 Expression BeadChips (Illumina, Inc., San Diego, CA, USA, catalogue number BD-103-0603) according to manufacturer's protocols. Microarrays were scanned with the Bead Array Reader (Illumina, Inc., San Diego, CA, USA).

### qPCR procedures

For validation of the results, cDNA was synthesized from 500 ng total RNA using Superscript II Reverse Transcriptase (Invitrogen, Darmstadt, Germany). qPCR was performed using the Universal Probe Library for TOMM7, RAB37, FKBP5, IL18R1 and TBP on the Roche LightCycler 480 (Roche Applied Science, Penzberg, Germany). Assays were designed using the Probe Finder Software (Roche Applied Science) and run in triplicates in a total reaction volume of 10 μl.

### Statistical analysis

Microarray expression analysis was performed in R [16], using *beadarray *(providing routines to handle Illumina BeadStudio data) [17], *limma *(for statistical routines) and *vsn *(for normalization) [18] packages. Raw microarray scan files were exported using the Illumina Beadstudio program and loaded into R for downstream analysis. The data were transformed and normalized using the variance stabilizing normalization method. Of the 48804 probes present on the Human HT-12v3, probes were selected which fulfilled the criteria of Illumina probe detection p-value of < 0.01 in the individuals. Raw qPCR data from the Roche LightCycler 480 System was extracted and the crossing thresholds (C_T_) from the technical replicates were averaged across all samples. Transcripts were normalized to housekeeping gene TBP.

Significantly regulated genes were detected using generalized linear models and corresponding fold changes between the groups in R. For differential expression analysis, functions from the limma package [19] were used on the normalized log_2_-transformed expression values before and after dexamethasone challenge; group differences were tested for collection method, globin reduction status, and pre- vs. post dexamethasone stimulation. Results were corrected for multiple testing by 10,000 permutations for each transcript using the permutation of regressor residuals test (PRR) as implemented in the R package glmperm (http://cran.r-project.org/web/packages/glmperm/index.html). Clinical variables and RNA quality measures were compared using a one way ANOVA with SigmaStat^® ^for Windows (Release 2.0, SPSS Inc., Chicago, Illinois 60606, USA).

## Abbreviations

RIN: RNA integrity number; CRP: C-reactive protein; GGT: Gamma glutamyl transpeptidase; ALAT: Alanine aminotransferase; ASAT: Aspartate aminotransferase.

## Competing interests

The authors declare that they have no competing interests.

## Authors' contributions

AM, EBB and DM participated in the design of the study and drafted the manuscript. MRH and TK performed the experiments; AM collected the samples; DM performed the statistical analysis; All authors read and approved the final manuscript.

## Disclosures

Monika Rex-Haffner; Torsten Klengel; Divya Mehta: nothing to disclose.

## Patents

Andreas Menke, Elisabeth B. Binder - inventors:

Means and methods for diagnosing predisposition for treatment emergent suicidal ideation (TESI). European application number: 08016477.5. International application number: PCT/EP2009/061575.

Elisabeth B. Binder - inventor:

FKBP5: a novel target for antidepressant therapy. International publication number: WO 2005/054500

Polymorphisms in ABCB1 associated with a lack of clinical response to medicaments. International application number: PCT/EP2005/005194

## Supplementary Material

Additional file 1 **Figure S1**. qPCR validation of TOMM7 and RAB37 baseline gene expression differences between PAXgeneTM and TempusTM tubes: Boxplots of TOMM7 and RAB37 expression levels from PAXgene™ and Tempus™ tubes are depicted. In accordance with microarray data, both transcripts were significantly differentially expressed between the PAXgene™ and Tempus™ tubes. Click here for file
